# Pathology of myelin oligodendrocyte glycoprotein antibody-associated disease: a comparison with multiple sclerosis and aquaporin 4 antibody-positive neuromyelitis optica spectrum disorders

**DOI:** 10.3389/fneur.2023.1209749

**Published:** 2023-07-21

**Authors:** Yoshiki Takai, Tatsuro Misu, Kazuo Fujihara, Masashi Aoki

**Affiliations:** ^1^Department of Neurology, Tohoku University Graduate School of Medicine, Sendai, Japan; ^2^Department of Multiple Sclerosis Therapeutics, Fukushima Medical University, Fukushima, Japan

**Keywords:** myelin oligodendrocyte glycoprotein, antibody, acute disseminated encephalomyelitis, perivenous demyelination, confluent demyelination, multiple sclerosis lesion pattern-II

## Abstract

Myelin oligodendrocyte glycoprotein (MOG) is expressed on the outermost layer of the myelin sheath in the central nervous system. Recently, the clinical concept of MOG antibody-associated disease (MOGAD) was established based on the results of human MOG-transfected cell-based assays which can detect conformation-sensitive antibodies against MOG. In this review, we summarized the pathological findings of MOGAD and discussed the issues that remain unresolved. MOGAD pathology is principally inflammatory demyelination without astrocyte destruction, characterized by perivenous demyelination previously reported in acute disseminated encephalomyelitis and by its fusion pattern localized in both the white and gray matter, but not by radially expanding confluent demyelination typically seen in multiple sclerosis (MS). Some of demyelinating lesions in MOGAD show severe loss of MOG staining compared with those of other myelin proteins, suggesting a MOG-targeted pathology in the disease. Perivascular cuffings mainly consist of macrophages and T cells with CD4-dominancy, which is also different from CD8+ T-cell-dominant inflammation in MS. Compared to aquaporin 4 (AQP4) antibody-positive neuromyelitis optica spectrum disorders (NMOSD), perivenous complement deposition is less common, but can be seen on myelinated fibers and on myelin degradation products within macrophages, resembling MS Pattern II pathology. Thus, the pathogenetic contribution of complements in MOGAD is still debatable. Together, these pathological features in MOGAD are clearly different from those of MS and AQP4 antibody-positive NMOSD, suggesting that MOGAD is an independent autoimmune demyelinating disease entity. Further research is needed to clarify the exact pathomechanisms of demyelination and how the pathophysiology relates to the clinical phenotype and symptoms leading to disability in MOGAD patients.

## 1. Introduction

Myelin oligodendrocyte glycoprotein (MOG) is a glycoprotein (consisting of 218 amino acids) expressed in oligodendrocytes and is characterized by its distribution in the outermost layer of the myelin sheath (1). MOG is composed of multiple splicing variants (2, 3), all of which have extracellular immunoglobulin variable domains and thus belong to the immunoglobulin superfamily (4). Because of these structural features, MOG has a long history of research as an autoantigen that can induce inflammatory demyelinating pathology in the central nervous system (CNS) (5–9), and is one of the best-studied antigens in experimental autoimmune encephalomyelitis (EAE) (10–12). Therefore, autoantibodies against MOG have long been considered a potential cause of human inflammatory demyelinating diseases, particularly multiple sclerosis (MS). However, the discovery of clinically relevant MOG antibodies in human disease has not been successful until recently. Previous results on the detection of MOG antibodies by enzyme-linked immunosorbent assay (ELISA) or Western blot were confusing due to the low specificity (13). This is because the antigen is linear in ELISA or denatured in Western blot such that the three-dimensional structure of native MOG was lost; the issue was resolved when the conformation-sensitive MOG antibody became detectable by human MOG-transfected cell-based assays (CBAs) (14–16). As a result, MOG antibodies have been found in patients with optic neuritis, acute myelitis, neuromyelitis optica spectrum disorders (NMOSD) without aquaporin 4 (AQP4) antibodies (17, 18), acute disseminated encephalomyelitis (ADEM) (19, 20), and brainstem (21–23) and cerebral cortical encephalitis (24–26). In contrast, typical MS patients are essentially negative for MOG antibodies (27, 28). Consequently, patients with MOG antibodies came to be recognized as belonging to a group with inflammatory demyelinating conditions distinct from MS, and the international diagnostic criteria of MOG antibody-associated disease (MOGAD) were recently published (29).

In this review, we summarized the histopathological findings of MOGAD in published studies. In particular, we outlined the pathologies typically found in MOGAD and the issues that remain unresolved because of inconsistent results in previous studies. We also discussed the unique pathogenesis of MOGAD by comparing it with MS and AQP4 antibody-positive NMOSD (AQP4 + NMOSD).

## 2. Histopathological features of MOGAD

### 2.1. Patterns of demyelination

**Figure 1:**
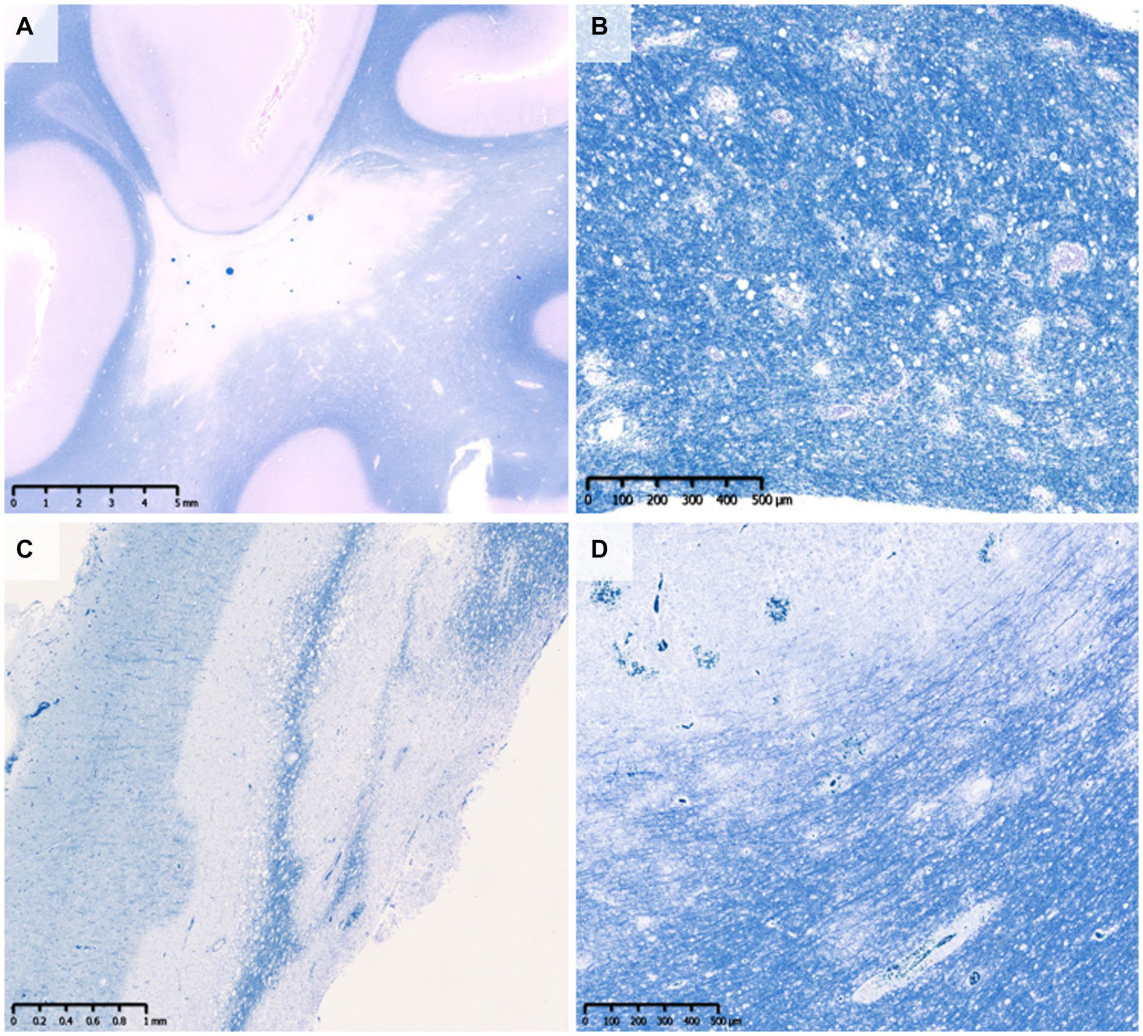
Various types of demyelination. **(A)** Confluent demyelination (SPMS). **(B)** Perivenous demyelination (ADEM). **(C)** Concentric demyelination (Balo’s disease). **(D)** Mixed pathology of confluent and perivenous demyelination (MOGAD). **(A–D)** Klüver-Barrera staining. ADEM, acute disseminated encephalomyelitis; MOGAD, myelin oligodendrocyte glycoprotein antibody-associated disease; SPMS, secondary progressive multiple sclerosis.

**Figure 2:**
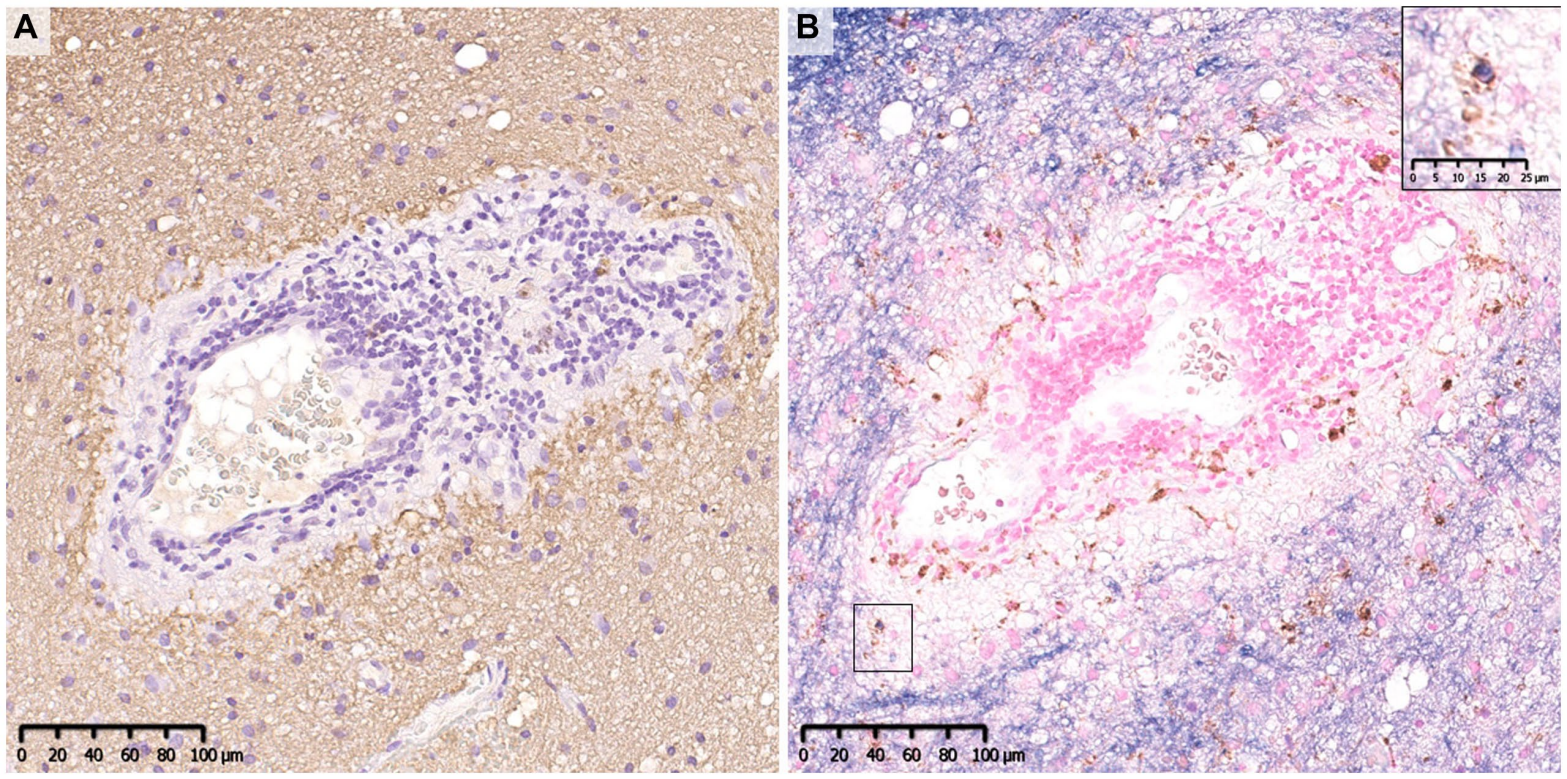
Perivenous demyelination in MOGAD. A demyelinating lesion was seen around small vessels with inflammatory cell infiltration. There were a small number of macrophages that phagocytosed myelin debris (insert in **B**). **(A)** MBP, **(B)** MOG (blue)/CD68 (brown). MBP, myelin basic protein; MOG, myelin oligodendrocyte glycoprotein; MOGAD, MOG antibody-associated disease.

The pattern of demyelination seen in well-known inflammatory demyelinating diseases can be classified into “confluent demyelination” in MS, “perivenous demyelination” in ADEM and “concentric demyelination” in Balo’s disease (Figure 1) (30, 31). “Confluent demyelination” is characterized by fusion and enlargement of perivascular demyelinating lesions with well-defined borders, resulting in the formation of large plaques, and the lesions may occasionally exhibit a map-like morphology (Figure 1A). On the other hand, “perivenous demyelination” is the one with indistinct borders around a single small vessel with inflammatory cell infiltration, and often multifocal (Figure 1B). Perivenous demyelination is considered useful in the pathological differentiation of ADEM from MS (32). However, it should be noted that we may miss “perivenous demyelination” because it can be very small, and the activity of myelin phagocytosis by macrophages is sometimes scarce, requiring careful observation (Figure 2).

**Figure 3:**
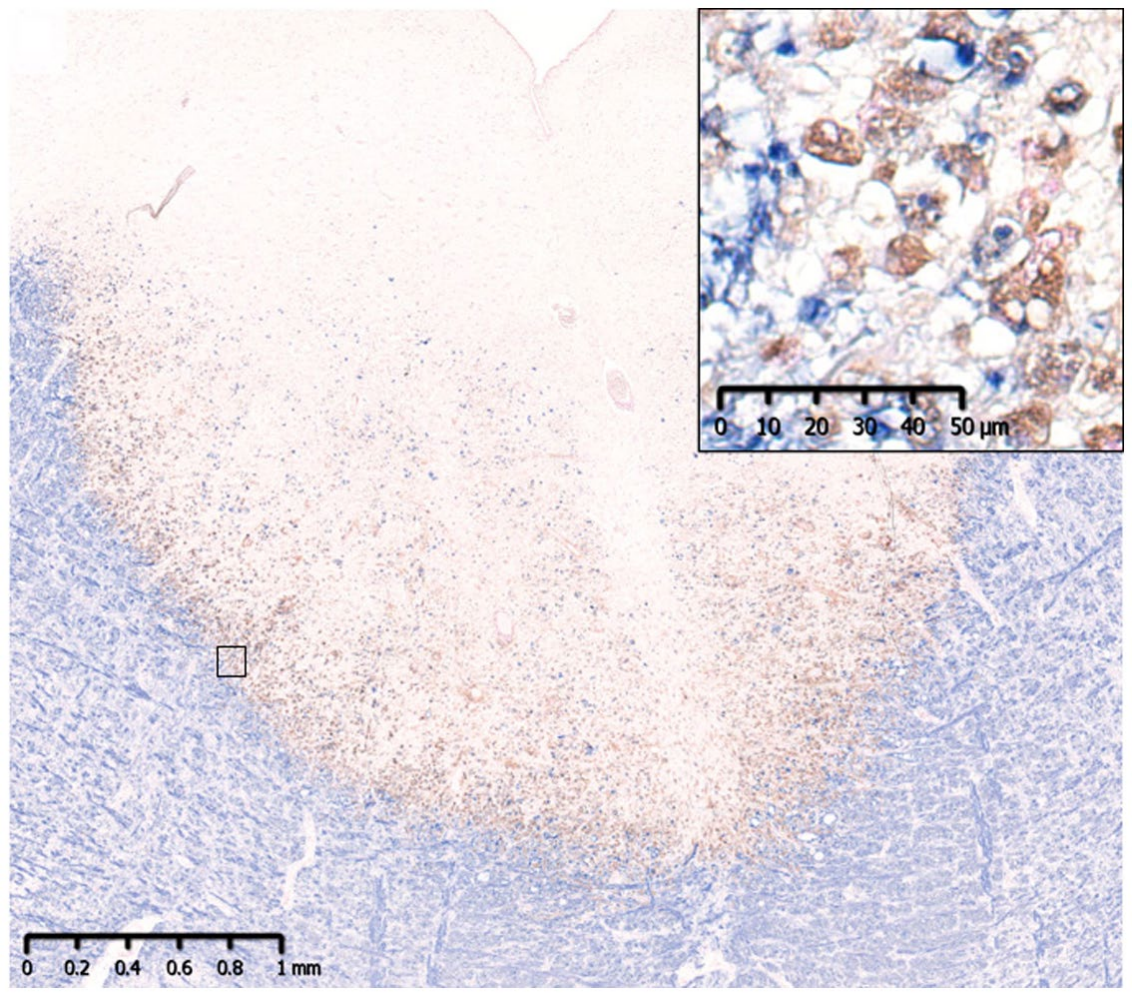
Slowly expanding lesion in SPMS. A large well-demarcated demyelinating lesion was seen with peripheral infiltration of myelin phagocytosed macrophages (insert). MBP (blue)/CD68 (brown). MBP, myelin basic protein; SPMS, secondary progressive multiple sclerosis.

The pathology of MOGAD is characterized by a mixture of perivenous and confluent demyelinating lesions (Figure 1D) (33, 34), and their proportions may depend on the timing of tissue sampling and disease severity (Tables 1, 2) (24, 33–47). The median time to tissue sampling in our study (one month) (33) was shorter than that in Höftberger et al. (seven months) (34) (Table 1), and the demyelination patterns in the two studies were different (90% of the lesions in our cases had perivenous demyelination, while 50% of the lesions in Höftberger’s study had a transitional pattern [a combination of perivenous and confluent demyelinations]). However, it is important to mention that among confluent demyelinating lesions, slowly expanding lesions (SELs) typically seen in the subacute to chronic stage of MS (Figure 3) (48) are rarely observed in MOGAD patients (34) or AQP4 + NMOSD patients (49). SELs are characterized by the accumulation of activated macrophages/microglia at the lesion edge with iron deposition (50), which is thought to be involved in the progression of MS (51–53). In other words, demyelinating lesion formation in MOGAD patients is characterized by simultaneous development of multiple perivascular inflammatory demyelination and its fusion to form confluent demyelination, which is different from radial expansion of the lesions in MS.

**Table 1:** Comparison of clinical findings and pathology of MOGAD in two studies of more than 10 patients.

Reference		Takai et al. (33)	Höftberger et al. (34)
Clinical findings	Patients, *n*	11	24 (Autopsy 2)
	Age (year)	29 (9–64)*	10 (1–66)*
	Female/Male (female %)	6/11, 55%	13/22, 59%
	Diagnosis	ADEM-like: 6/11 LE: 3/11 CCE: 2/11	ADEM-like: 11/18 NMOSD: 1/18 Myelitis: 2/18 ON: 2/18 CCE: 1/18 BS: 1/18
	Time from attack to biopsy (month)	1 (0.5–96)*	7 (0–516)*
	Total follow up period (month)	33 (12–180)*	43 (3–516)*
Pathology	Demyelination pattern		
	Perivenous (ADEM-like)	91%	21%
	Confluent (MS-like)	2%	29%
	Transitional (perivenous + confluent)	7%	50%
	MOG-dominant myelin loss	37%	0%
	Astrocytopathy	0/11 (0%)	0/17 (0%)
	CD4-dominant T-cell infiltration	10/11 (91%)	+
	Complement deposition	2/11 (18%)	8/8 (100%)

*Median (range). ADEM, acute demyelinating encephalomyelitis; BS, brain stem lesion; CCE, cortical encephalitis; LE, leukoencephalopathy; NMOSD, neuromyelitis optica spectrum disorders; MOG, myelin oligodendrocyte glycoprotein; MOGAD, MOG antibody-associated disease; MS, multiple sclerosis; ON, optic neuritis.

**Table 2:** Summary of the clinical and pathological findings of MOGAD in case reports.

A					
Clinical findings					Reference
Case	Age	Sex	Clinical phenotype	Antibody other than MOG	Author
1	49	F	Rel.TDL (open ring)	nr	Konig et al. (35)
2	71	M	Blt.ON, MY, multiple brain lesions	AQP4	Di Pauli et al. (36)
3	66	F	Rel.myelitis + TDL (open ring)	-	Spadaro et al. (37)
4	63	F	CIS	-	Jarius et al. (38)
5	67	F	Rel.LETM + TDL (multiple)	-	Wang et al. (39)
6	49	M	ADEM (MY + multiple brain lesion)	nr	Körtvélyessy et al. (40)
7	34	M	ADEM (MY + multiple brain lesion)	nr	
8	28	F	Blt.ON + TDL (infiltrative)	-	Zhou et al. (41)
9	25	F	ADEM + ON	-	
10	29	F	CCE + Blt.ON	-	Ikeda et al. (42)
11	46	M	CCE + ON	-	Fujimori et al. (24)
12	47	M	ADEM (diffuse)	-	Komatsu et al. (43)
13	45	M	TDL (infiltrative)	-	Shu et al. (44)
14	6	F	TDL (infiltrative)	-	
15	37	F	CCE + MY	-	Papathanasiou et al. (45)
16	40	M	CCE + multiple brain/brain stem lesion	P/C-ANCA	
17	52	F	TDL (lymphoma)	-	Uzura et al. (46)
18	17	M	CCE + ON + MY	-	Valencia-Sanchez et al. (47)
19	35	F	CCE	-	

B											
Pathological findings											
Case	Material	Demyelination pattern	Oligodendrocyte	Damaged myelin component	Astrocytopathy		Site of complement deposition			IgG deposition	Infiltrating inflammatory cells
					Astrocyte morphology	AQP4-loss	Myelin	Inside macrophage	Perivascular		
1	Biopsy (Brain)	Nr	Nr	Nr	Nr	Nr	Nr	+	Nr	Nr	M, T
2	Autopsy	Confluent	Preoligodendrocyte	MOG = MBP > CNP ase	Loss	+	Nr	Nr	+	Nr	CD3, CD8, low B,
3	Biopsy (Brain)	Confluent	Preserved	MOG > PLP	Reactive	−	+	+	−	Diffuse with fiber	M, T (CD8)
4	Biopsy (Brain)	Nr	Preserved	Even	Reactive	−	Nr	+	Nr	Macrophage	CD4 = CD8, B
5	Biopsy (Brain)	Confluent	Nr	Nr	Reactive	Nr	Nr	Nr	Nr	Nr	M, T, low B
6	Biopsy (Brain)	Confluent	Apoptosis	MAG > MOG	Nr	Nr	+	+	Nr	+	M,T (CD8), B
7	Biopsy (Brain)	Confluent	Preserved	Even	Nr	Nr	Nr	Nr	+	Nr	M (rim), T dominant, CD8
8	Biopsy (Brain)	Confluent	Nr	Nr	Reactive	−	Nr	Nr	Nr	Nr	M, T (CD4), low B
9	Biopsy (Brain)										
10	Biopsy (Brain)	Perivenous, subpial	Preserved	MOG > MAG, MBP	Reactive	−	−	−	−	−	M, T (CD4 > CD8), low B
11	Biopsy (Brain)	No demyelination	Nr	Nr	Nr	Nr	Nr	Nr	Nr	Nr	M, T, B
12	Biopsy (Brain)	Perivenous, confluent	Preserved	MOG > MAG, MBP	Reactive	−	+	+	−	Diffuse	M, T (CD4 > CD8), low B
13	Biopsy (Brain)	Confluent	Preoligodendrocyte	Nr	Reactive	Decrease	−	Minor	−	Nr	M, T (CD4 > CD8), low B
14	Biopsy (Brain)	Confluent	Preoligodendrocyte	Nr	Reactive	Decrease	−	Minor	−	Nr	M, T (CD4 > CD8), low B
15	Biopsy (Brain)	Nr	Nr	Nr	Nr	Nr	Nr	Nr	Nr	Nr	M, T, B
16	Biopsy (Brain)	Perivenous, confluent	Nr	Nr	Nr	Nr	Nr	Nr	Nr	Nr	T, B
17	Biopsy (Brain)	Perivenous, confluent	Preserved	MOG > MAG, MBP	Reactive	−	−	−	−	Perivenous	M, T, low B
18	Biopsy (Brain)	Perivenous, subpial	Nr	even	Nr	Nr	−	−	−	Nr	M, T (CD4 > 8)
19	Biopsy (Brain)	Subpial	Nr	Nr	Nr	Nr	Nr	Nr	Nr	Nr	M, T (CD4 = 8), B*

(A) ADEM, acute disseminated encephalomyelitis; ANCA, anti-neutrophil cytoplasmic antibody; AQP4, aquaporin 4; Blt., bilateral; CCE, cerebral cortical encephalitis; CIS, clinically isolated syndrome; F, female; LETM, longitudinally extensive transvers myelitis; M, male; MOGAD, myelin oligodendrocyte glycoprotein antibody-associated disease; MY, myelitis; ON, optic neuritis; P/C, perinuclear/cytoplasmic; Rel., relapsing; TDL, tumefactive demyelinating lesion. *, **Same patient in case11* and case7** in reference (33). (B) Nr, not reported; MAG, myelin associated glycoprotein; MOG, myelin oligodendrocyte glycoprotein; MBP, myelin basic protein; CNP, 2′,3’-Cyclic-nucleotide 3′-phosphodiesterase; PLP, Proteolipid protein; M, macrophage; T, T cells; B, B cells. +, present; −, absent. *Focal meningeal B-cell agglutination without features of ectopic B-cell follicles.

### 2.2. Distribution of demyelinating lesions

**Figure 4:**
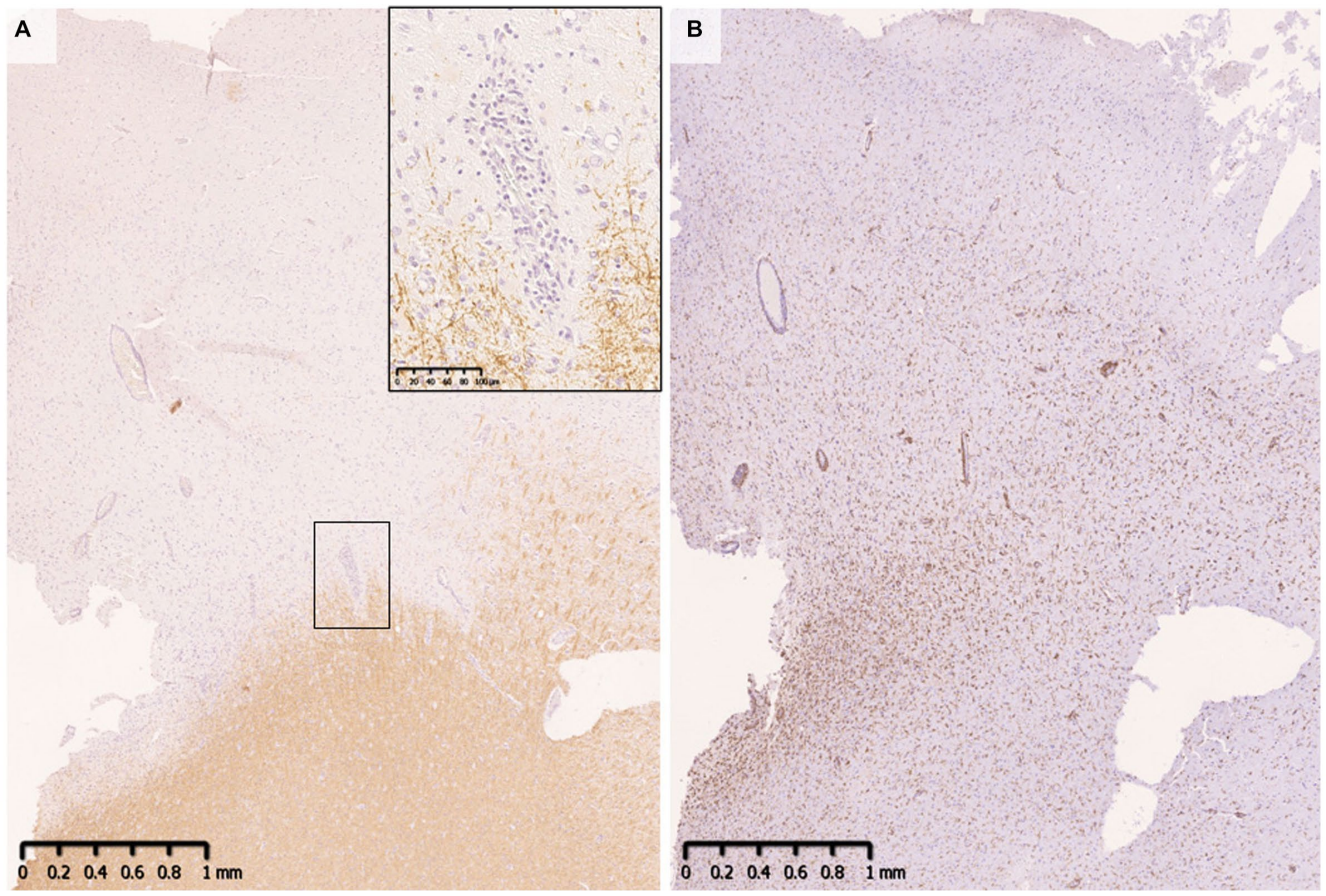
Subpial demyelination in MOGAD. **(A)** Myelin fibers were widely lost in the subpial cortex. Perivenous demyelination was observed at the cortical-medullary junction. **(B)** CD68 positive macrophages/microglia were diffusely infiltrated the demyelinated cortex. **(A)** MBP, **(B)** CD68. MBP, myelin basic protein; MOGAD, myelin oligodendrocyte glycoprotein antibody-associated disease.

Demyelinating lesions in MOGAD patients are found mainly in the white matter but also in the subpial cortex to cortico-medullary junction and deep gray matter (Figure 4) (33, 34, 47). Within these lesions, CD68-positive macrophages/microglia widely infiltrate the cortex (Figure 4). The frequency of cortical demyelination is reported to be higher in MOGAD patients with cerebral involvement than in MS patients (34), which is compatible with the high incidence of cortical involvement in MOGAD patients, evidenced by conditions such as ADEM and cerebral cortical encephalitis (29). In addition, inflammatory cells infiltrate around meningeal vessels adjacent to subpial demyelinating lesions (33, 34, 45, 47). In cerebral cortical encephalitis of MOGAD patients, brain MRI scans often show contrast enhancing effects in the meninges which may reflect such inflammation around the meningeal vessels (47).

### 2.3. Preferential loss of specific myelin component(s)

**Figure 5:**
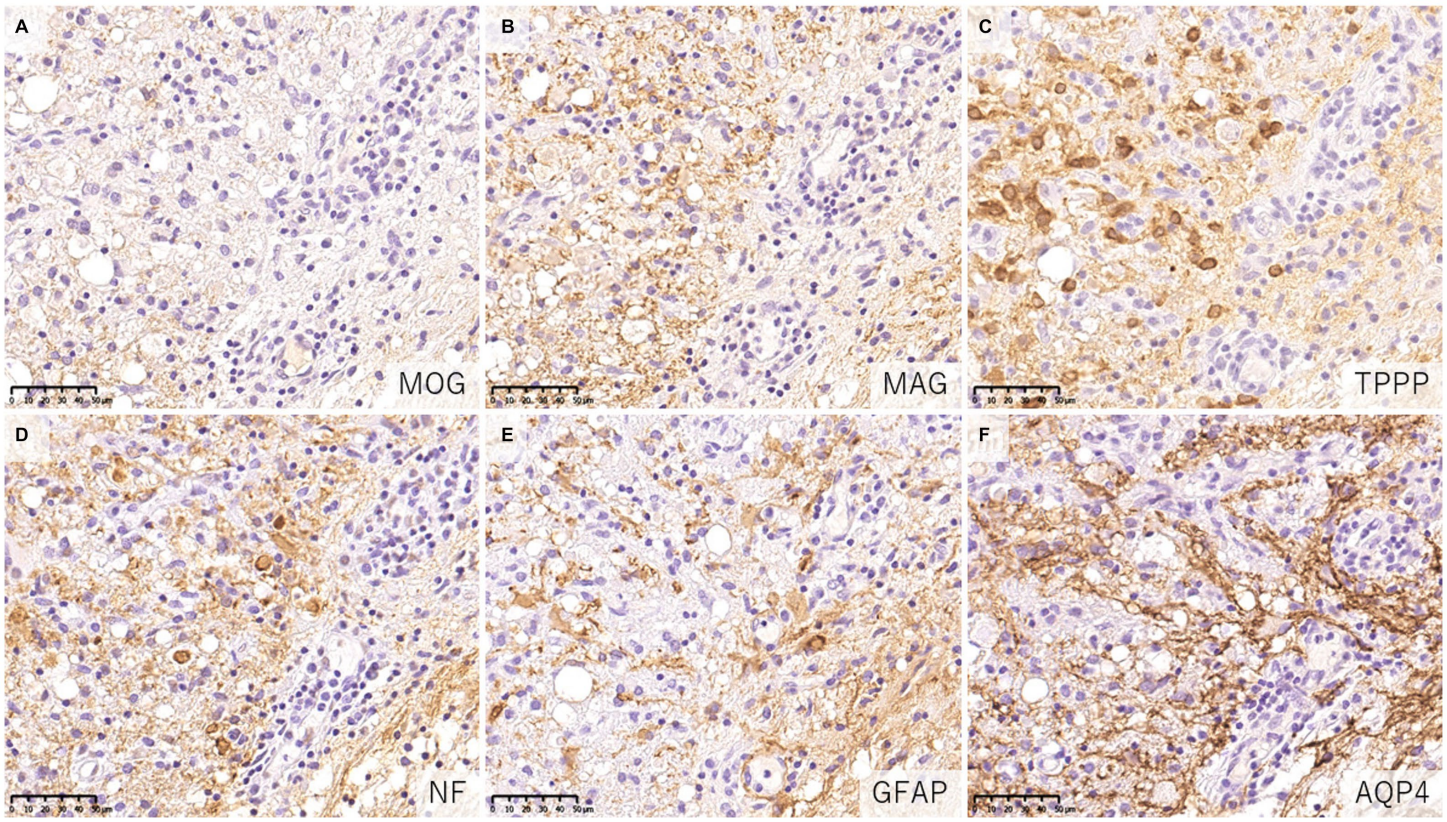
Characteristics of demyelinating lesions in MOGAD. **(A,B)** Loss of MOG staining was more evident than MAG staining. **(C)** Oligodendrocytes were well preserved in the demyelinating lesion. **(D)** Axonal enlargement was present, suggesting neuroaxonal alteration but axonal staining was relatively preserved compared to demyelination. **(E,F)** Activated astrocytes with dense AQP4 staining were observed. **(A)** MOG, **(B)** MAG, **(C)** TPPP, **(D)** NF, **(E)** GFAP, **(F)** AQP4. AQP4, aquaporin 4; GFAP, glial fibrillary acidic protein; MAG, myelin associated glycoprotein; MBP, myelin basic protein; MOG, myelin oligodendrocyte glycoprotein; MOGAD, MOG antibody-associated disease; NF, neurofilament; TPPP, tubulin polymerization promoting proteins.

When evaluating demyelinating lesions, it is important to identify primarily damaged myelin component(s) by immunohistochemistry to assess the type and stage of the disease (48, 54). Our study and several previous case reports indicated that some demyelinating lesions found in patients with MOGAD showed MOG-dominant myelin loss especially in the early-stage (Figure 5) (33, 37, 42, 46), and some myelin-laden macrophages localized at the perivascular space showed MOG-dominant phagocytosis (33). In addition, oligodendrocytes are relatively preserved in MOGAD demyelinating lesions (33, 37, 38, 44). These findings support that MOGAD actually targets MOG and that its demyelination process may initially occur on the surface of the myelin sheath. However, Höftberger et al. reported no MOG dominant myelin loss in their study (34), and it remains to be clarified what this difference originated from. On the other hand, some reports indicated that preferential loss of myelin associated glycoprotein (MAG) could occur in MOGAD patients although the incidence was very low (34, 40). Since MAG is expressed in the innermost layer of the myelin sheath and is most distant from the oligodendrocyte cell body, preferential loss of MAG is thought to reflect oligodendrocyte damage (distal oligodendrogliopathy) (55). This finding is seen in patients with MS pattern III lesions (56); Balo’s disease (57); AQP4 + NMOSD (58); and ischemic tissue damage such as cerebral infarction (59). Since MOG is also expressed at the surface of oligodendrocytes, depending on the concentration or characteristics of MOG antibodies, some oligodendrocytes may be damaged by MOG antibodies.

### 2.4. Characteristics of inflammatory cell infiltration

The cellular infiltrate in inflammatory demyelinating lesions is composed mainly of myelin phagocytosing macrophages at the sites of demyelination and T-cell clusters in the perivascular space (perivascular cuffing). Infiltrating cells in the lesions of MOGAD are essentially similar (Tables 1, 2), but a characteristic feature of MOGAD is CD4+ T-cell-dominant infiltration in the demyelinating lesions (33, 34), which is different from the dominance of CD8+ T-cell infiltrates in MS lesions (60, 61). Some of those CD4+ T cells in MOGAD might be reactive to MOG epitopes. However, it should be noted that the timing of the sampling of specimens should be considered. In AQP4 + NMOSD, the main subpopulation of T cells infiltrated in the lesions changes from CD4 in the acute phase to CD8 in the chronic phase of the disease (49). Most of the CNS tissue specimens of MOGAD patients examined in the published studies were obtained in the acute phase, and the pathological findings in the chronic phase have not been examined. Therefore, the characteristics of T cells infiltrating the lesion may reflect differences in the stage of the disease rather than pathogenesis, and further detailed verification is required in the future. However, it is known that the levels of T helper 17 (Th17)-related cytokines are markedly elevated in the cerebrospinal fluid (CSF) of patients during the acute phase of MOGAD and AQP4 + NMOSD when compared with those of MS patients and control subjects (62). Thus, in the acute phase, T-cell subpopulations infiltrating the lesions are different between MOGAD and MS patients.

B cells are seen in small numbers in the perivascular space, but are less frequent than T cells (33, 34). Additionally, ectopic lymphoid follicles, as reported in MS (63, 64), have not been detected in MOGAD patients. However, occasionally B-cell aggregates may be seen in the leptomeninges (47). In MOGAD patients, intrathecal production of MOG antibodies seems to occur more frequently than in AQP4 + NMOSD patients (65–68), suggesting that B cells infiltrating the CNS produce MOG antibodies and contribute to the pathogenesis of the disease. In addition, CXCR4 is upregulated in B cells in patients with MOGAD (69), and its ligand, CXCL12, is known to be elevated in the CSF (70). Thus, CXCL12/CXCR4 may contribute to the chemotaxis of B cells and other inflammatory cells in MOGAD patients (71).

### 2.5. Deposition of humoral immunity and complement activity

Since MOG antibodies are mainly composed of those in the IgG1 subclass (27, 72), complement-mediated cytotoxicity (CDC) has been considered to contribute to the pathogenesis of MOGAD. In fact, both *in vitro* and *in vivo*, it has been reported that MOG antibody-induced cytotoxicity and demyelination can occur in a complement-mediated manner (72–74). Indeed, some previous case reports on biopsied brain lesions in MOGAD patients showed the deposition of complement components on myelin fibers and myelin debris phagocytosed by macrophages, and the authors concluded that the lesions were probably caused by humoral immune-mediated demyelination, such as MS pattern II lesions (37, 38, 40, 56). This type of MS lesion is histologically characterized by extensive confluent demyelination with tissue deposition of humoral immune factors such as complements and immunoglobulins (22). However, the histopathological findings of complements in MOGAD patients remain debatable, as the reported results have been inconsistent (33, 34). In our study, only 2 of 11 MOGAD patients showed tissue deposition of complement, which was much less frequent and dense than in AQP4 + NMOSD patients with perivascular deposition of activated complements (C9neo) in all acute lesions (33). However, Höftberger et al. concluded that active complement deposition was observed in all 8 patients they evaluated (34) (Table 2). This difference may be due to the clinical severity, timing of tissue sampling or inter-individual variability in the severity of MOG-IgG-related cytotoxicity other than complement activation, such as antibody-dependent cellular phagocytosis (ADCP) and antibody-dependent cellular cytotoxicity (ADCC) (75, 76). However, a recent *in vitro* study demonstrated that MOG antibodies elicited much less complement activation than AQP4 antibodies (77). AQP4 has two isoforms, M1 and M23, that differ in their transcription start sites (78). AQP4-M1 and M23 are coexpressed in the CNS, and M23 is known to form large well-ordered assemblies called orthogonal arrays of particles (OAPs) (78) and is reported to be more highly expressed in the optic nerve and spinal cord, where NMOSD lesions are more likely to occur (79). The formation of OAPs allows AQP4 to be densely expressed on the cell surface, facilitating AQP4 antibody clustering on the cell membrane. The classical complement activation pathway is initiated by the binding of C1q to the Fc portion of IgG, but requires bivalent or multivalent binding (80). Thus, complement components are more likely to be activated when IgG is densely bound on the plasma membrane, and in fact, the presence of OAPs significantly enhanced complement-mediated cytotoxicity by the presence of AQP4 antibodies (81). On the other hand, MOG constitutes a quantitatively minor component (0·05%) of the myelin sheath (1), and MOG antibodies require bivalent binding when binding to MOG, making it difficult for them to assemble on the cell membrane, and resulting in low C1q binding ability (82). It is necessary to study in detail whether the amount and characteristics of MOG antibodies affect the degree of complement activation following binding to AQP4.

## 3. Comparison between MOGAD and MS

The dominant pattern of demyelinating lesions (perivenous demyelination) in MOGAD is similar to that of ADEM rather than MS. The characteristics of infiltrating T cells also differ in MOGAD and MS, as noted above (Table 3). However, we cannot rule out the possibility that some patients with MOGAD may have a pathology similar to MS since previous studies on MOG-EAE have demonstrated that the ratios of myelin antigen-specific lymphocytes and autoantibodies to myelin could influence the dominance of perivenous or confluent demyelination (8, 85). Additionally, there is a report that MOG antibodies purified from two MOGAD patients (whose MOG antibodies were capable of binding to rodent MOG) and administered intrathecally to EAE subjects induced by MOG-specific T cells did not produce demyelinating lesions with deposition of activated complement, but in the presence of MBP-specific T cells, demyelinating lesions similar to MS pattern II developed (86). These findings suggested that T cells in some cases of MOGAD may recognize myelin protein(s) other than MOG and activate complements. Thus, further investigations are needed to confirm whether this is the case in the human pathology of MOGAD. However, it should be noted that MS Pattern II is a pathological classification proposed before the discovery of conformation-sensitive MOG antibodies and includes many brain biopsy samples from cases with atypical or fulminant cerebral lesions for MS (56). Jarius et al. found that only one of the 13 cases with MS Pattern II pathology was positive for MOG antibodies and suggested its limited involvement (38). Therefore, it may include other inflammatory demyelinating pathologies than MS and MOGAD and require further verification.

**Table 3:** Comparison of the major pathological findings of acute lesions in MOGAD, MS, and AQP4 + NMOSD.

Disease	MOGAD	MS	AQP4 + NMOSD
Primary target	Myelin > Oligodendrocyte	Myelin, Oligodendrocyte	Astrocyte
Histopathology			
Lesion distribution	Mainly in WM, the cerebral cortex and deep GM can also be involved	Mainly in periventricular and juxtacortical WM, (cerebral cortex in the progressive phase)	Both WM and GM, mainly in the spinal cord and optic nerves
Pattern of demyelination	Perivenous > Confluent or Transitional*	Confluent (SEL in the progressive phase)	Secondary in the astrocyte lytic lesions, Distal oligodendrogliopathy
Lesion edge	Ill-defined ~ sharply defined	Sharply defined	Sharply defined
Damaged myelin proteins	MOG > or = others	MAG > others (in Pattern III) or Even (in the other patterns)	MAG > others
Oligodendrocyte	Relatively preserved	Partially loss ~ regenerate	Loss
Astrocyte	Reactive	Reactive	Loss
AQP4-loss	None ~ Mild	None ~ Mild	Severe
Axon	Preserved	Relatively preserved, (degenerated in the progressive phase)	Damaged in various degrees
Site of complement deposition	Myelin, inside macrophage	Myelin, inside macrophage (in MS Pattern II)	Vasculocentric (rim/rosette pattern)
Cellular infiltration			
Macrophage	Most conspicuous in the PVS and parenchyma	Most conspicuous in parenchyma, especially at the lesion edge	Most conspicuous in the PVS and parenchyma
T cells	CD4 dominant in the PVS	CD8 dominant in the PVS	CD4 dominant in the PVS (CD8 dominant in the chronic phase)
B cells	A small number in the PVS, occasional aggregates in the leptomeninges	A small number in the PVS (Ectopic lymphoid follicles in the progressive phase)	A small number in the PVS
Neutrophil/Eosinophil	Mild ~ Moderate	Rare	Mild ~ Marked
Fluid pathology			
Cell damage marker	MBP elevated, GFAP not elevated	MBP elevated, GFAP not elevated (elevated in the progressive phase) (83, 84)	MBP elevated, GFAP remarkably elevated
Cytokine profile	Marked elevation of Th17-related cytokines relative to MS		Marked elevation of Th17-related cytokines relative to MS

AQP4, aquaporin 4; GFAP, glial fibrillary acidic protein; GM, gray matter; MAG, myelin associated glycoprotein; MBP, myelin basic protein; MOG, myelin oligodendrocyte glycoprotein; MOGAD, MOG antibody-associated disease; MS, multiple sclerosis; NMOSD, neuromyelitis optica spectrum disorders; PVS, perivascular space; SEL, slowly expanding lesion; WM, white matter. *Perivenous + Confluent.

## 4. Comparison between MOGAD and AQP4 + NMOSD

**Figure 6:**
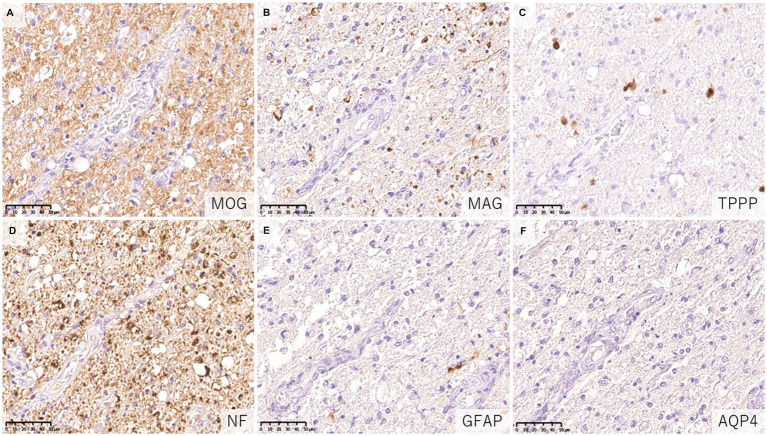
Characteristics of astrocytopathic lesions in AQP4 + NMOSD. **(A,B)** Loss of MAG staining was more evident than MOG staining. **(C)** Numerous oligodendrocytes were lost in the lesion. **(D)** Axonal enlargement was present, suggesting neuroaxonal alteration, but axonal staining was relatively preserved compared to demyelination and astrocyte loss. **(D–F)** Astrocytes were almost completely lost in the lesion. **(A)** MOG, **(B)** MAG, **(C)** TPPP, **(D)** NF, **(E)** GFAP, **(F)** AQP4. AQP4: aquaporin 4, GFAP, glial fibrillary acidic protein; MAG, myelin associated glycoprotein; MBP, myelin basic protein; MOG, myelin oligodendrocyte glycoprotein; MOGAD, MOG antibody-associated disease; NF, neurofilament; NMOSD, neuromyelitis optica spectrum disorders; TPPP, tubulin polymerization promoting proteins.

**Figure 7:**
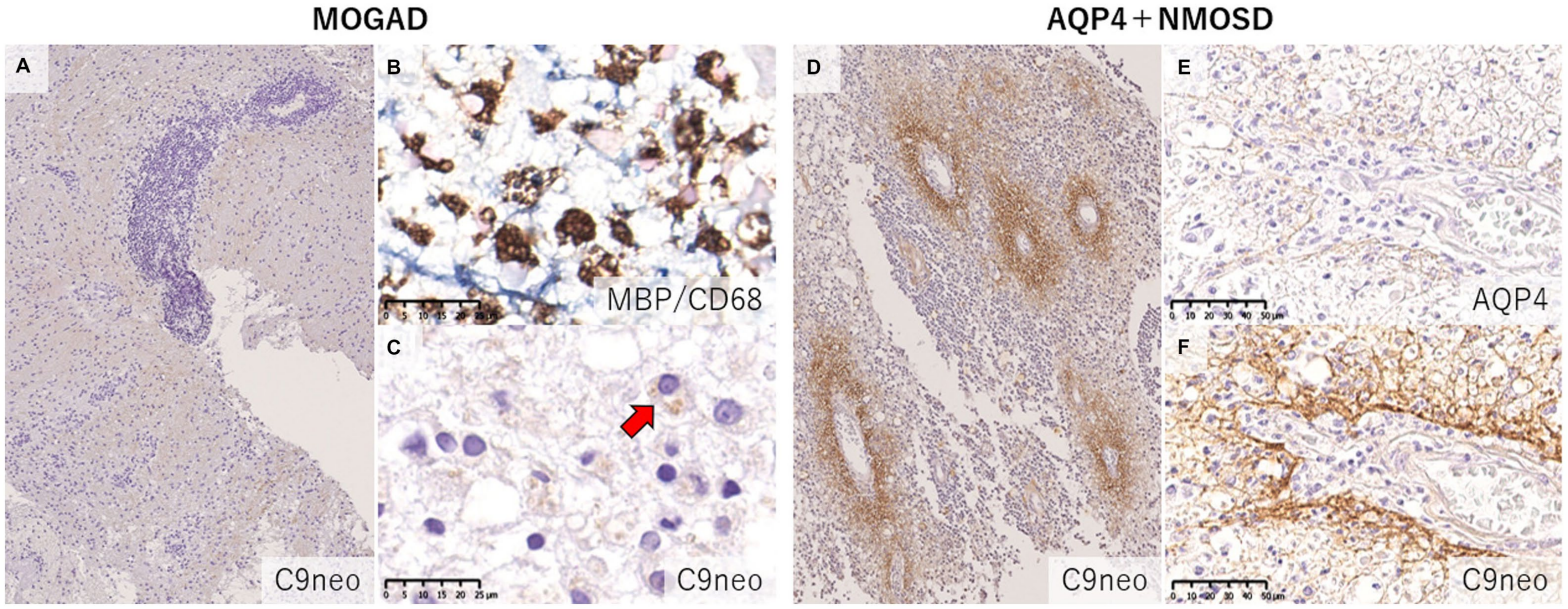
Comparison of the deposition pattern of activated complements in MOGAD and AQP4 + NMOSD. **(A)** Only mild perivascular depositions of complements were seen in MOGAD even in the active lesion where perivascular cuffing was evident. **(B,C)** In active demyelinating lesions, complement staining was detected on myelin debris phagocytosed by macrophages (red arrow). **(D)** Multiple rosette-like stainings of complement deposition were seen in the NMOSD lesion. **(D–F)** Perivascular complement depositions were seen within AQP4-loss lesions. **(A,C,D,F)** C9neo. **(B)** MBP/CD68, **(E)** AQP4. AQP4, aquaporin 4; MBP, myelin basic protein; MOGAD, myelin oligodendrocyte glycoprotein antibody-associated disease; NMOSD, neuromyelitis optica spectrum disorders.

The fundamental difference in the pathologies of the two diseases is that the main target of immune attack is myelin in MOGAD but is astrocytes in AQP4 + NMOSD (62). In previous pathological studies of MOGAD, there has been no astrocytic damage except in patient doubly positive for AQP4 and MOG antibodies (36). In the demyelinating lesions in MOGAD, astrocytes are essentially activated and AQP4 is also strongly stained on immunohistochemistry images (Figure 5), although two cases of partially decreased AQP4 expression in MOGAD with tumefactive brain lesions have been reported (Table 2) (44). The pathological process starts in the perivascular regions in both MOGAD and AQP4 + NMOSD, but they show distinct features of demyelinating lesions: in MOGAD, MOG is predominantly lost with relatively preserved oligodendrocytes, whereas in AQP4 + NMOSD, MAG is preferentially damaged, and oligodendrocytes are lost, but MOG is relatively preserved (Figure 6). The immunohistochemical staining pattern of activated complement deposition also differs: a rosette-like staining around blood vessels is seen in AQP4 + NMOSD (49, 87, 88), while in MOGAD, perivascular complement deposition is much less (Figure 7) but stained on myelinated fibers and in myelin degradation products within macrophages (Figure 7; Tables 2, 3) (37, 38, 40). Despite these different patterns of demyelination, MOGAD and AQP4 + NMOSD share some clinical features, such as optic neuritis and longitudinally extensive myelitis (18, 89), and cytokine profiles (upregulation of Th17-related cytokines) in the CSF (62) as autoantibody-associated CNS diseases.

## 5. Conclusion

Recently, the international diagnostic criteria for MOGAD have been proposed (29), and certain pathological features of the disease have been clarified (29), indicating that MOGAD is a disease entity distinct from MS and AQP4 + NMOSD. In fact, published articles on MOGAD have been rapidly increasing in recent years, and a few international clinical trials for relapsing MOGAD have already begun.

Considering the currently available data of the histopathological studies of MOGAD and some basic research with MOG antibodies, the immunopathological process in MOGAD may be summarized as follows. Initially, the breakdown of immune tolerance leads to the generation of MOG-reactive T cells that stimulate the production of MOG antibodies from B cells in the periphery. Triggered by infection, vaccination, or other stimuli, these MOG-reactive T cells are activated and penetrate the blood–brain barrier (BBB) into the CNS and aggregate at the perivascular space of the meninges and parenchyma (perivascular cuffing). MOG antigens in the CNS further activate these cells which are primarily CD4-positive T cells and promote a Th17-dominant cytokine milieu in the CNS and the BBB disruption. As a result, more MOG antibodies enter the CNS. Then the autoantibodies target myelins, especially MOG, to demyelinate the nerve fibers from the surface of myelin sheath via CDC (noted as deposition of activated complements), ADCC (in cooperation with infiltrating granulocytes), ADCP (seen as myelin phagocytosed macrophages), and other mechanisms. A fraction of the demyelinating lesions may exhibit MOG-dominant loss, suggesting a MOG-targeted pathology, and some oligodendrocytes may also be damaged. But compared to the remarkable CDC to cause astrocytolysis in AQP4 + NMOSD, the pathological role of CDC for demyelination may be less in some cases of MOGAD. These events probably occur simultaneously around multiple blood vessels (perivenous demyelination) in the white and gray matters. Subsequently, broken MOG and other myelin components are phagocytosed by macrophages (myelin-laden macrophages in the parenchyma and perivascular space), further enhancing antigen presentation and activating MOG-reactive CD4-positive T cells that induce the activation and infiltration of cytotoxic effector T cells against myelins and B cells that produce MOG antibodies intrathecally. These cellular and humoral immune responses are augmented through the interaction with proinflammatory cytokines/chemokines, which further exacerbates the disease state resulting in fusion of the lesions to form extensive demyelination (confluent demyelination). This confluent demyelination in MOGAD may develop by a different mechanism from that of the radial expansion of MS lesions.

However, we should investigate further details of the pathophysiology of MOGAD by means of various technologies including molecular immunology, omics, advanced imaging, neurophysiological tests, therapeutic response and artificial intelligence as well as conventional histopathological analyses. Furthermore, the histopathological studies in MOGAD to date have been derived from brain biopsies, and we should clarify whether the lesion characteristics are similar in other CNS regions, such as the optic nerve and spinal cord. Studies on how differences in the histopathologic findings may affect the severity and clinical phenotype in patients with MOGAD are also needed. These studies are expected to contribute to a better understanding and management of MOGAD.

## Author contributions

YT, KF, and MA contributed to conception and design of the study. YT and TM organized the database. YT wrote the first draft of the manuscript, tables, and figures. All authors contributed to manuscript revision, read, and approved the submitted version.

## Conflict of interest

The authors declare that the research was conducted in the absence of any commercial or financial relationships that could be construed as a potential conflict of interest.

## Publisher’s note

All claims expressed in this article are solely those of the authors and do not necessarily represent those of their affiliated organizations, or those of the publisher, the editors and the reviewers. Any product that may be evaluated in this article, or claim that may be made by its manufacturer, is not guaranteed or endorsed by the publisher.
